# Book Review

**DOI:** 10.1120/jacmp.v12i1.3428

**Published:** 2010-09-14

**Authors:** Luke McLemore

**Affiliations:** ^1^ Department of Radiation Oncology Mayo Clinic 200 First Street SW Rochester MN 55905 USA

## Abstract

*The Checklist Manifesto: How to Get Things Right* by Atul Gawande, M.D. Metropolitan Books, Henry Hold and Company, LLC, 2009, ISBN 978‐0‐8050‐9174‐8

The current climate, one in which patient safety is on the forefront of the minds and agendas of many within the medical physics community as well as others affiliated with our occupation, presents an opportune time to read a book that holds the promise of getting things right in a complex world.

Dr. Atul Gawande, the author of the book under review, is a general and endocrine surgeon at Brigham and Women's Hospital, an associate professor at Harvard Medical School and the Harvard School of Public Health, and is the director of the World Health Organization's Safe Surgery Saves Lives program. He is also author of *Better: A Surgeon's Notes on Performance* (Picador, 2007) and *Complications: A Surgeon's Notes on an Imperfect Science* (Picador, 2002).

In the introduction of *The Checklist Manifesto: How to Get Things Right*, Atul Gawande poses the question: Why, in the realm of things that are within the reach of our control and where we have tremendous knowhow, training and expertise, do we fail at what we set out to do? His answer is that science has created complexity, which causes enormous strains on humans in making good on the promise it affords. The solution, he proclaims, is that checklists are what one needs to overcome failure and achieve success in the modern era.

My response to this proposal, at first, echoes that of other skeptics. It seems all too easy, simplistic and almost absurd to think that checklists are going to be “the solution”. Anticipating this skeptical view, Gawande takes a very systematic approach to present his case in defense of the checklist. He argues that despite best efforts to manage complexity in medicine through providing more training, specialization and expertise, medical professionals have not been successful in preventing mistakes. While his examples and illustrations focus more on surgery, medical physicists can certainly relate to the dilemma of continued mistakes in our field despite increased efforts in training, expertise and specialization.

Using many intriguing examples and stories from a myriad of disciplines such as aviation, medicine, construction, the restaurant business and the entertainment industry, he continues to build his case by revealing how checklists have been – and are being – successfully used to manage complexity. He shows how checklists can help with some of the more obvious items such as memory recall and establishing sets of “minimum necessary steps” for processes. In addition, he also points out capabilities of checklists that one may overlook. For example, how checklists are able to “establish a higher standard of baseline performance”, “catch mental flaws”, establish and guide communication, build teamwork, incorporate and assign responsibility, be used as a “behavior change vehicle”, and many others.

The World Health Organization's global initiative to reduce “avoidable deaths and harm from surgery” is Gawande's primary example and mandate. In the book, he shares how he and other distinguished medical professionals from around the globe wrestled to discover a viable and affordable solution to this complex problem. Elaborating on the journey that led WHO to embrace a checklist, he gives captivating insights into the challenges that came with it. A checklist, though simple in concept and form, is anything but trivial when it comes to its development and implementation, and Gawande turns to the aviation industry for guidance. Incorporating what he learns, he candidly discusses some of the trials and tribulations encountered during the refining and test phases of the checklist's construction. The most challenging aspects of the exercise, he says, are deciding which items to include on the checklist, and maintaining balance between “brevity and effectiveness”.

The development and testing of the WHO safe surgery checklist, which contained nineteen items broken up into three separate “pause” points, is well covered. The results of WHO's piloting the checklist in eight diverse hospitals around the world are nothing short of astonishing, and are published in the January 2009 issue of *The New England Journal of Medicine*.^(^ ^1^ ^)^

Gawande concludes the book with final arguments to combat the resistance and fears to using checklists, followed by some personal examples to give the reader glimpses of his own journey with the WHO safe surgery checklist. He presents what he believes is the underlying reason of why we do not like checklists, and seeks to motivate the reader and the medical community to rise to the opportunity to embrace them. In his personal stories, he shares how his own skepticism towards the usefulness of the checklist is transformed into belief. In the final example, Gawande tells how using the WHO safe surgery checklist during one of his surgeries saved his patient's life.

As physicists, we use checklists often. One may use checklists as reminders of what monthly QA tests need to be completed, what steps to take in performing QA tests, or what items to verify prior to an HDR treatment, SRS treatment, etc. However, are we using them in ways Gawande propose they should be used? That is, are we attempting to devise our checklists so that they not only serve as memory reminders or means by which to outline sets of steps to follow, but they also incorporate team building, establish communication, and assist in averting errors? Have we broadened our horizons and considered the utility of checklists in areas of our practice that perhaps we may have never considered? For instance, could a “pause” with some basic verbal checks immediately prior to every patient's treatment be of benefit? The book naturally leads one to ponder questions such as these. Thankfully, Gawande provides practical guidance on creating quality checklists.

Is the checklist “the solution” to managing complexity and ensuring success? Gawande's zeal to share his newfound passion for the checklist is an obvious bias; he does not give much attention to other tools that may be just as useful. For instance, as physicists we are very familiar with thoughtful implementation and use of interlocks. Circumventing an error through the removal or restriction of an opportunity that leads to the error is an exercise that indubitably leads to achieving success. Additional examples include the role that human factors engineering and systems engineering should play as we move forward in our quest to minimize risk and the occurrence of error while providing high‐quality care. Dr. Gawande alludes to these topics throughout the book; however, he does not capitalize on their usefulness nor stress that checklists are but one weapon in the arsenal that one can and should utilize, perhaps in combination, to achieve success and avert errors in complex environments.

In summary, the book is fascinating, an easy read, and presents very practical information for application. One can easily read the book while flying to an AAPM meeting or listen to it during daily commute to work. The book, recommended to me, is one I recommend to you. I do not think it will change your life, but it clearly indicates that checklists hold the promise of being tools, when utilized properly, that may lead one to further success while navigating complexity and, furthermore, may save the life of one of your patients.
